# Data safe havens to combine health and genomic data: benefits and challenges

**DOI:** 10.23889/ijpds.v1i1.348

**Published:** 2017-04-19

**Authors:** Kerina H Jones, Arron S Lacey, Brian L Perkins, Mark I Rees

**Affiliations:** 1 Swansea University

## Objectives

Data safe havens can bring together and combine a rich array of anonymised person-based data for research and policy evaluation within a secure setting. To date, the majority of available datasets have been structured micro-data derived from routine health-related records. Possibilities are opening up for the greater reuse of genomic data such as Genome Wide Association studies (GWAS) and Whole Exome/Genome Sequencing (WES or WGS). However, there are considerable challenges to be addressed if the benefits of using these data in combination with health-related data are to be realized safely. 

## Approach

We explore the benefits and challenges of using genomic datasets with health-related data, and using the Secure Anonymised Information Linkage (SAIL) system as a case study, the implications and way forward for Data Safe Havens in seeking to incorporate genomic data for use with health-related data. 

## Results

The benefits of using GWAS, WES and WGS data in conjunction with health-related data include the potential to explore genetics at a population level and open up novel research areas. These include the ability to increasingly stratify and personalize how medical indications are detected and treated through precision medicine by understanding rare conditions and adding socioeconomic and environmental context to genomic data. Among the challenges are: data availability, computing capacity, technical solutions, legal and regulatory frameworks, public perceptions, individual privacy and organizational risk. Many of the challenges within these areas are common to person-based data in general, and often Data Safe Havens have been designed to address these. But there are also aspects of these challenges, and other challenges, specific to genomic data. These include issues due to the unknown clinical significance of genomic information now or in the future, with corresponding risks for privacy and impact on individuals. 

## Conclusion

Genomic data sets contain vast amounts of valuable information, some of which is currently undefined, but which may have direct bearing on individual health at some point. The use of these data in combination with health-related data has the potential to bring great benefits, better clinical trial stratification, epidemiology project design and clinical improvements. It is, therefore, essential that such data are surrounded by a properly-designed, robust governance framework including technical and procedural access controls that enable the data to be used safely.

